# Bed Utilisation in an Irish Regional Paediatric Unit – A Cross-Sectional Study Using the Paediatric Appropriateness Evaluation Protocol (PAEP)

**DOI:** 10.15171/ijhpm.2016.53

**Published:** 2016-05-10

**Authors:** Coilín ÓhAiseadha, Mai Mannix, Jean Saunders, Roy K. Philip

**Affiliations:** ^1^Department of Public Health, Health Service Executive, Dublin, Ireland.; ^2^Statistical Consulting Unit, University of Limerick, Limerick, Ireland.; ^3^Regional Paediatric Unit (Children’s Ark), University Hospital Limerick (UHL), Limerick, Ireland.

**Keywords:** Bed Utilisation, Bed Occupancy, Hospitalisation, Paediatrics, Quality Of Healthcare, Social Work, Clinical Audit

## Abstract

**Background:** Increasing demand for limited healthcare resources raises questions about appropriate use of inpatient beds. In the first paediatric bed utilisation study at a regional university centre in Ireland, we conducted a cross-sectional study to audit the utilisation of inpatient beds at the Regional Paediatric Unit (RPU) in University Hospital Limerick (UHL), Limerick, Ireland and also examined hospital activity data, to make recommendations for optimal use of inpatient resources.

**Methods:** We used a questionnaire based on the paediatric appropriateness evaluation protocol (PAEP), modified and validated for use in the United Kingdom, to prospectively gather data regarding reasons for admission and for ongoing care after 2 days, from case records for all inpatients during 11 days in February (winter) and 7 days in May–June (summer). We conducted bivariate and multivariate analysis to explore associations between failure to meet PAEP criteria and patient attributes including age, gender, admission outside of office hours, arrival by ambulance, and private health insurance. Inpatient bed occupancy and day ward activity were also scrutinised.

**Results:** Mean bed occupancy was 84.1%. In all, 12/355 (3.4%, 95% CI: 1.5%–5.3%) of children failed to meet PAEP admission criteria, and 27/189 (14.3%, 95% CI: 9.3%–19.3%) who were still inpatients after 2 days failed to meet criteria for ongoing care. 35/355 (9.9%, 95% CI: 6.8%–13.0%) of admissions fulfilled only the PAEP criterion for intravenous medications or fluid replacement. A logistic regression model constructed by forward selection identified a significant association between failure to meet PAEP criteria for ongoing care 2 days after admission and admission during office hours (08.00–17.59) (*P* = .020), and a marginally significant association between this outcome and arrival by ambulance (*P* = .054).

**Conclusion:** At a mean bed occupancy of 84.1%, an Irish RPU can achieve 96.6% appropriate admissions. Although almost all inpatients met PAEP criteria, improvements could be made regarding emergency access to social services, management of parental anxiety, and optimisation of access to community-based services. Potential ways to provide nasogastric or intravenous fluid therapy on an ambulatory basis, and outpatient antimicrobial therapy (OPAT) should be explored. Elective surgical admissions should adhere to day-of-surgery admissions (DOSA) policy.

## Background

Concerns about the adverse effects of high bed occupancy^1^ and increasing demand for limited healthcare resources raise questions about appropriate use of inpatient beds,^2^ which may be assessed using the bed utilisation review.

The paediatric appropriateness evaluation protocol (PAEP) was derived from the appropriateness evaluation protocol (AEP) for adult inpatient care in the United States,^3^ modified and validated for use in the United Kingdom.^4,5^ The protocol is used to evaluate appropriateness of admission and of continuing inpatient care on another day, here designated the “day of care.” Appropriateness of admission is assessed according to criteria for (A) severity of illness and (B) intensity of service.^5^ Appropriateness of continuing care is assessed according to criteria for (A) medical services, (B) nursing/life support services and (C) patient condition.

International studies using the original or modified versions of the PAEP found rates of inappropriate bed utilisation of 2.0%–40.7% at the time of admission,^6-12^ and 21.4%–55.5% on subsequent days.^10,11,13,14^ Greater rates of inappropriate admission were found in association with attributes including male gender,^9^ daytime admission,^10^ and distant residence.^11^ Greater rates of inappropriate continuing inpatient care were associated with factors such as female gender,^10^ age >5 years,^14^ medical versus surgical admission,^10,11^ and inappropriate admission.^10,11^

We used the PAEP to assess the utilisation of inpatient beds at the Regional Paediatric Unit (RPU) in University Hospital Limerick (UHL), and also examined hospital activity data, with a view to recommendations to optimise the use of inpatient resources.

The RPU includes two inpatient wards, Rainbow Ward and Sunshine Ward, and also Caterpillar Day Ward. Rainbow Ward accommodates infants and toddlers up to the age of 15 months, with a capacity of 23 beds. The paediatric high-dependency unit (PHDU) with paediatric critical care level II facilities has an approved operational capacity of 2 inpatients. Nursing complements are reallocated from Rainbow Ward when PHDU is in use. Sunshine Ward accommodates children aged 15 months to 14 years, with a capacity of 27 beds. These two wards together receive approximately 5000 admissions per year, with an average length of stay of 2.82 days. Caterpillar Day Ward, with a capacity of 7 beds, serves nearly 5000 day cases annually.

The main route of inpatient admissions is the regional emergency department (ED), where approximately 15000 children under 14 years are assessed annually.

### Rationale

The study was triggered by an informal observation of high bed occupancy, with sustained, intense demands on staff, particularly those responsible for inpatient care. It was considered appropriate to review available data with a view to determining whether the RPU’s practices were compliant with international best practice, and whether improvements could be made to balance the best patient care with optimal use of scarce resources.

### Aim

The over-arching aim of the study was to audit our inpatient paediatric activity against the modified PAEP criteria and identify measures to make the most effective use of limited acute paediatric inpatient bed stock.

## Methods

This study was conducted as (*i*) an audit of compliance of paediatric inpatient care with PAEP criteria and (*ii*) a review of paediatric activity in the RPU. The study did not extend to the care of adult patients.

### Audit Tool

We used a PAEP-based study tool to collect information on demographics and attributes including age, gender, date and time of admission, private/public health insurance, referral source, presenting diagnosis and discharge planning as well as PAEP criteria. (Public health insurance is provided to holders of a “medical card” issued on the basis of weekly income below a certain threshold.) Questions were inserted to record the views of consultants regarding admissions which might be found not to fulfil the criteria, through structured interview. Questions asked whether the consultant considered that the child merited admission or ongoing care, and requested a reason or reasons for admission or delayed discharge.

One of the assessors had been trained in the use of a PAEP-based study tool in association with a previous bed utilisation study, and provided training to the second assessor during a pilot study for this study. Both assessors had gained thorough familiarity with operational issues in paediatric units and with paediatric case records through previous experience as non-consultant hospital doctors (NCHDs).

### Sample Size

In preparation for the study, the assessors reviewed the literature and conducted a pilot study involving a small sample of ten inpatients. An earlier, unpublished Irish study found that 9.8%–10.2% of paediatric patients failed to meet criteria on the day of admission (DOA), and 19.6%–34.7% failed to meet criteria on the day of care (Downey and Bedford, 2008). In our pilot study, 3/10 (30%) children were found not to meet PAEP criteria on admission. It was anticipated that, if even 20% of admissions failed to meet PAEP criteria, then it would be desirable and feasible to strive to reduce the proportion to a target of 10% on later re-audit. To detect a statistically significant reduction in the proportion of inappropriate admissions from 20% to 10%, with 90% power, the required sample size in each group is 266. This would also be sufficient if the baseline proportion were greater, eg, 30%, as suggested by the pilot study. Accordingly, a total sample size of at least 266 admissions was considered appropriate.

###  Data Collection

Data were gathered for all inpatients admitted or receiving ongoing care during two study periods, one of 11 consecutive days in February 2013 (winter season) and another of 7 consecutive days in May–June 2013, including two days of the June bank holiday weekend (summer season).

The PAEP admission criteria were applied to each child at the time of admission. With a view to comparability of inter-subject results, the PAEP criteria for ongoing care were applied to each child on an index day, 2 days after admission. To validate data extraction processes, inter-observer concordance was tested by comparison of independently completed forms for a random sample of one in every 10 admissions.

Discharge planning was considered to be documented for any patient whose medical or nursing records indicated a projected date of discharge or any preparations for discharge.

The assessors jointly conducted a structured face-to-face interview with the admitting consultant regarding each child who did not meet PAEP criteria on the DOA or day of ongoing care (DOC), as outlined above.

All recorded data were entered into an Excel® spreadsheet and independently validated before import into SPSS® for analysis.

###  Inclusion and Exclusion

The PAEP admission criteria were applied to all new admissions during the two study periods. The PAEP criteria for ongoing care were applied to each inpatient still receiving inpatient care 2 days after admission. No patients were excluded from the study.

### Statistical Analysis

Bivariate and multivariate analysis (using SPSS® 21.0) was used to test for associations between failure to meet PAEP admission criteria and age, gender, county of residence, specialty, health insurance status, admission outside of office hours (08.00–17.59), weekend admission, source of referral, elective/emergency admission or arrival by ambulance, and for associations between failure to meet PAEP day-of-care criteria and age, gender, county of residence, specialty, source of referral, health insurance, discharge planning or failure to meet admission criteria.

As most of the independent variables were dichotomous, or could meaningfully be recoded as dichotomous variables, bivariate analysis was conducted by chi-square test and, when the expected frequency for any cell in a 2×2 contingency table was <5, by Fisher exact test. Age was recoded into the categories <15 months or ≥15 months, in accordance with the age threshold for admission to Sunshine Ward rather than Rainbow Ward. As the RPU is centrally situated within Limerick city and county, county of residence was recoded as Limerick versus any other, more remote county. DOA was recoded as weekend or weekday. Source of referral was recoded as self/other, to test the hypothesis that the outcomes of self-referral might differ from those of referral from general practitioner (GP) or other professional sources.

Multivariate analysis was conducted by attempting to construct a logistic regression model for each of the two outcomes of interest: (*i*) failure to meet PAEP criteria on admission; and (*ii*) failure to meet PAEP criteria on reassessment 2 days after admission. In each instance, the approach was forward selection, with variables being selected if they made a significant improvement to the model.

### Hospital Activity Data

Data for inpatient bed occupancy and day ward activity were tabulated and reviewed.

## Results

### Sample Achieved

The study captured data for 355 admissions, 211/355 (59.3%) in the 11 days February 18–28, 2013 (winter season), and 144/355 (40.7%) in the 7 days May 27–June 2, 2013 (summer season). Of these, 189/355 (53.2%) of children were still receiving inpatient care 2 days after admission.

Although the assessors recorded different primary diagnoses for 2/36 (5.6%) of admissions, due to multiple recorded differential diagnoses, independent concordance was achieved for 36/36 (100%) in all other fields.

### Descriptive Statistics

The distribution by gender and age group of children admitted is presented in Table 1. Males were in a slight majority, constituting 53.2% of all admissions, while females constituted 46.8%. Almost a quarter were aged under 1 year, and almost three-fifths (212/355) were aged under 5 years. Because age 15 months was used as the threshold for admission to Sunshine Ward rather than Rainbow Ward, the distribution between age groups under 15 months and 15 months or over is presented in Table 2, along with a summary of other attributes of the admissions in the sample.

**Table 1:** Age Group and Gender Distribution of Children Admitted

	**Female**		**Male**		**Total**	
	**n**	**%**	**n**	**%**	**n**	**%**
<1 year	35	9.9	53	14.9	88	24.8
>5 years	75	21.1	68	19.2	143	40.3
1-4 years	56	15.8	68	19.2	124	34.9
Grand Total	166	46.8	189	53.2	355	100.0

**Table 2:** Descriptive Statistics for Study Sample at Admission and in Ongoing Care 2 Days Later

Inpatient Attribute	** DOA**		**DOC **	
	**n**	**%**	**n**	**%**
Gender				
Female	166	46.8	89	47.1
Male	189	53.2	100	52.9
Age			** **	
Under 15 months	100	28.2	57	30.2
15 months or over	255	71.8	132	69.8
Place of residence			** **	
Limerick city/county	205	57.8	105	55.5
County Clare	108	30.4	65	34.4
Other	42	11.8	19	10.1
Health insurance				
Private insurance only	130	36.6	76	40.2
Medical card only	138	38.9	72	38.1
Private insurance and medical card	20	5.6	11	5.8
Neither	67	18.9	30	15.9
Season				
Winter	211	59.4	117	61.9
Summer	144	40.6	72	38.1
DOA				
Weekend (Saturday/Sunday)	107	30.1	58	30.7
Weekday (Monday–Friday)	248	69.9	131	69.3
Time of admission				
Office hours (08.00 am–17.59 pm)	181	52.8	106	57.6
Out of hours (18.00 pm–07.59 am)	162	47.2	78	42.4
Specialty				
Medical	273	76.9	156	82.5
Surgical	82	23.1	33	17.5
Type of admission				
Elective	27	7.6	6	3.2
Emergency	327	92.4	182	96.8
Source of referral				
GP	186	52.4	102	54.0
Self	97	27.3	63	33.3
Other/unknown	72	20.3	24	12.7
Arrival by ambulance?				
Yes	18	5.1	12	6.3
No	321	90.4	166	87.8
Unknown	16	4.5	11	5.9

Abbreviations: DOA, day of admission; DOC, day of ongoing care; GP, general practitioner.

Almost twice as many children admitted were resident in County Limerick (205/355, or 57.7%) as in the adjacent County Clare (108/355, or 30.4%), and only 29/355 (8.2%) of admitted children were resident in County Tipperary, while 13/355 (3.7%) were resident elsewhere.

Since 130/355 (36.6%) of children had private health insurance only, and 20/355 (5.6%) had both private insurance and a medical card, a total of 150 (42.3%) were admitted as private patients, while 138 (38.9%) of admissions had a medical card and 67 (18.9%) had neither private health insurance nor a medical card.

A total of 273/355 (76.9%) were admitted as paediatric medical patients, and 81/355 (22.8%) were admitted as paediatric surgical patients. In all, 27/355 (7.6%) of admissions were elective: 19 surgical and 8 medical.

Sources of referral included 186/355 (52.4%) from GP, 97/355 (27.3%) self-referred. Other sources included 24/355 (6.8%) from outpatient department (OPD), 11/355 (3.1%) from another hospital, 13/355 (3.7%) from unknown sources and smaller numbers from various other sources such as day ward, social worker or school.

### Fulfilment of PAEP Criteria for Day of Admission

In all, 343 children (96.6%) met the PAEP criteria on admission, and 12 children (3.4%, 95% CI: 1.5%–5.3%) failed to meet the criteria (Table 3), with similar proportions in both specialties (two-tailed *P*=.310, Fisher exact test).

**Table 3:** Failure to Meet PAEP Criteria for DOA, by Specialty

**Admission Criteria Met?**	**Medical**		**Surgical**		**Both Specialties**	
	**n**	**%**	**n**	**%**	**n**	**%**
Yes	262	96.0	81	98.8	343	96.6
No	11	4.0	1	1.2	12	3.4
Total	273	100.0	82	100.0	355	100.0

Abbreviations: DOA, day of admission; PAEP, paediatric appropriateness evaluation protocol.

The distribution by other attributes of inpatients who did not meet admission criteria is summarised in Table 4, along with bivariate test statistics.

**Table 4:** Bivariate Tests for Associations Between Attributes of Admissions and Failure to Meet PAEP Admission Criteria

**Attribute of Admission**	**Did not Meet DOA Criteria**		**Total Admissions**	**Test Statistics**
	**n**	**%**	**N**		
Gender					
Female	6	3.6	166	χ² = 1.316; df = 1; *P* = .251.
Male	6	3.2	189	Fisher: *P* = 1.000.
Age group					
Under 15 months	3	3.0	100	χ² = 0.062; df = 1; *P* = .804.
15 months or over	9	3.5	255	Fisher: *P* = 1.000.
Place of residence					
Limerick city or county	5	2.4	205	χ² = 1.316; df = 1; *P* = .251.
Outside Limerick	7	4.7	150	Fisher: *P* = .373.
Health insurance				
Private health insurance	4	2.7	150	χ² = 0.405; df = 1; *P* = .525.
Medical card/no insurance	8	8.1	205	Fisher: *P* = .570.
Season					
Winter	9	4.3	211	χ² = 1.206; df = 1; *P* = .272.
Summer	3	2.1	144	Fisher: *P* = .374.
DOA					
Weekend (Saturday/Sunday)	2	1.9	107	χ² = 1.071; df = 1; *P* = .301.
Weekday (Monday–Friday)	10	4.0	248	Fisher: *P* = .522.
Time of admission					
Office hours (08.00 am – 17.59 pm)	7	3.9	181	χ² = 0.154; df = 1; *P* = .694.
Out of hours (18.00 pm – 07.59 am)	5	3.1	162	Fisher: *P* = .775.
Specialty					
Medical	11	4.0	273	χ² = 1.524; df = 1; *P* = .217.
Surgical	1	1.2	82	Fisher: *P* = .309.
Type of admission					
Elective	0	0.0	27	χ² = 1.026; df = 1; *P* = .311.
Emergency	12	3.7	327	Fisher: *P* = .610.
Source of referral					
Self	2	2.1	97	χ² = 0.710; df = 1; *P* = .399.
GP	10	3.9	258	Fisher: *P* = .524.
Arrival by ambulance?					
Yes	1	5.6	18	χ² = 0.451; df = 1; *P* = .502.
No	9	2.8	321	Fisher: *P* = .425.

Abbreviations: DOA, day of admission; PAEP, paediatric appropriateness evaluation protocol.

Of the 211 winter admissions, a total of 9 (4.3%) were outside PAEP criteria on DOA, consisting of 8 medical patients and 1 surgical patient. Of the 144 summer admissions, 3 (2.1%) medical patients and no surgical patients were outside the criteria. The seasonal difference was not statistically significant (Fisher exact test: *P*=.370).

For each of 343/355 (96.6%) of the admissions for whom time of admission was recorded in the chart, time of admission was categorised as “Office hours” or “Out of hours,” depending on whether the child was admitted between the hours of 08.00 am and 17.59 pm or not. There was no statistically significant difference in the distribution of admissions that failed to meet PAEP criteria according to whether they were admitted within or out of office hours (Fisher exact test: *P*=.780).

Of the 12 patients who failed to fulfil PAEP criteria for DOA, 4/12 (33.3%) had private health insurance, 5/12 (41.7%) had a medical card only and 3/12 (25.0%) had no record of either. There was no statistically significant difference in fulfilment of admission criteria between those with private insurance and those without (Fisher exact test: *P*=.570).

Bivariate analysis indicated no statistically significant association between any of the other independent variables, expressed as dichotomous categories, and the outcome of failure to meet PAEP admission criteria (Table 4).

In all, 159/355 (44.8%) of the admissions fulfilled criterion 4, “Intravenous medications and/or fluid replacement.” While 124/355 (34.9%) fulfilled another criterion also, 35/355 (9.9%, 95% CI: 6.8%–13.0%) fulfilled no other PAEP criterion.

When *multivariate analysis* was conducted to identify which patient attributes formed the best logistic regression model predicting the outcome of failure to meet admission criteria, none of the variables were found to be eligible for construction of a predictive model.

Of note, although 5 children were admitted for social reasons alone, only one of these failed to meet PAEP criteria. A social worker expressed fears for the safety of two children on the basis of a perception that their parent was not able to protect them while engaging in behaviours that might endanger them. Two more children were admitted when a member of the national police service, An Garda Síochána, expressed fears for their safety after violence had been committed to another member of the family. A fifth was admitted solely because one of the child’s parents was admitted acutely ill while the other parent was not immediately available to care for the child. The first 4 of these admissions were judged to meet PAEP criterion number 20, “Special paediatric problems: (A) Child abuse, due to severity of injuries or no safe place available,” but the fifth did not, because there was no actual violence or imminent threat of violence and thus no actual child abuse.

### Potential Alternatives and Reasons for Admission

Upon review of the case notes of those 12 patients who did not meet PAEP criteria, the assessors observed that 5 admissions could have been avoided through access to appropriate outpatient services, and 6 through care at home with the support of a GP, a clinical nurse specialist or a social worker. One child was admitted solely because his/her parent had been acutely admitted to hospital.

The children’s paediatricians indicated that 7 (58.3%) patients merited admission although they did not meet the criteria, for (not mutually exclusive) reasons including parental anxiety (5), unsuccessful treatment in the community (2), social issues (1), and clinical complexity (1).

### Fulfilment of Paediatric Appropriateness Evaluation Protocol Criteria for Day of Care

As 166/355 (46.8%) children had been discharged within 2 days (48 hours) of admission, proportions within PAEP criteria on the day of care were calculated using the denominator of 189 children who were still in hospital 2 days after admission. Of these, 162/189 (85.7%) children met the PAEP criteria, while 27/189 (14.3%) failed to meet the criteria.

When analysed by specialty (Table 5), 22/156 (14.1%) medical and 5/33 (15.2%) surgical admissions failed to meet the criteria (*P*=.790, Fisher exact test).

**Table 5:** Failure to Fulfil PAEP Criteria 2 Days After Admission, by Specialty

**PAEP Criteria Met? **	**Medical**		**Surgical**		**Both Specialties**	
	**n**	**%**	**n**	**%**	**n**	**%**
Yes	134	85.9	28	84.8	162	85.7
No	22	14.1	5	15.2	27	14.3
Total	156	100.0	33	100.0	189	100.0

Abbreviation: PAEP, paediatric appropriateness evaluation protocol.

The distribution by other attributes of inpatients who did not meet PAEP criteria for ongoing inpatient care 2 days after admission is summarised in Table 6, along with bivariate test statistics.

**Table 6:** Bivariate Tests DOC

**Attribute of Inpatient**	**Did not Meet DOC Criteria**		**Total Inpatients**	**Test Statistics**
	**n**	**%**	**N**	
Gender					
Female	11	12.4	89	χ² = 0.510; df = 1; *P* = .475.
Male	16	16.0	100	Fisher: *P* = .536.
Age					
Under 15 months	11	19.3	57	χ² = 1.675; df = 1; *P* = .196.
15 months or over	16	12.1	132	Fisher: *P* = .257.
Place of residence					
Limerick City/County	14	13.3	105	χ² = 0.175; df = 1; *P* = .676.
Outside Limerick	13	15.5	84	Fisher: *P* = .682.
Health insurance					
Private health insurance	9	10.3	87	χ² = 2.045; df = 1; *P* = .153.
Medical card/no insurance	18	15.3	102	Fisher: *P* = .211.
Season					
Winter	15	12.8	117	χ² = 0.538; df = 1; *P* = .463.
Summer	12	16.7	72	Fisher: *P* = .523.
Day of assessment					
Weekend (Saturday/Sunday)	10	17.2	58	χ² = 0.597; df = 1; *P* = .440.
Weekday (Monday–Friday)	17	13.0	131	Fisher: *P* = .500.
Time of admission^a^				OR = 0.358
Office hours (08.00–17.59)	20	18.9	106	χ² = 4.625; df = 1; *P* = .032.
Out of hours (18.00–07.59)	6	7.7	78	Fisher: *P* = .034.
Specialty					
Medical	22	14.1	156	χ² = 0.024; df = 1; *P* = .876.
Surgical	5	15.2	33	Fisher: *P* = .791.
Type of admission					
Elective	2	33.3	6	χ² = 1.814; df = 1; *P* = .178.
Emergency	25	13.7	182	Fisher: *P* = .207.
Source of referral					
Self	10	15.9	63	χ² = 0.194; df = 1; *P* = .659.
Other/unknown	17	13.5	126	Fisher: *P* = .664.
Arrival by ambulance?^a^				OR = 0.203
Yes	5	41.7	12	χ² = 7.554; df = 1; *P* = .006.
No	21	12.7	166	Fisher: *P* = .017.
Documented discharge planning?^a^					
Yes	12	15.0	80	χ² = 1.40; df = 1; *P* = .708.
No	14	13.1	109	Fisher: *P* = .831.
Failed to meet DOA criteria					
Yes	2	100.0	2	χ² = 12.128; df = 1; *P* < .001.
No	25	12.8	187	Fisher: *P* = .020.

Abbreviations: OR, Odds ratio; DOC, day of ongoing care. ^a^Because of missing data, not all totals for those who did not meet DOC criteria add up to 27.

Bivariate testing indicated a significant association between failure to meet the PAEP day-of-care criteria and admission within office hours. Those children who were admitted during office hours, ie, between 08.00 and 17.59, were less likely to meet the PAEP criteria for ongoing inpatient care two days later (odds ratio [OR]: 0.358; 95% CI: 0.137–0.940; *P*=.034, Fisher exact test). Children who arrived for admission by ambulance were likewise less likely to meet the PAEP criteria for ongoing care two days later (OR: 0.203; 95% CI: 0.059–0.698; *P*=.017, Fisher exact test).

In addition, there was a significant association between failure to meet PAEP criteria on the DOC and failure to meet admission criteria (*P*=.020, Fisher exact test). If patients failed to meet PAEP criteria for the DOA, they were also more likely to fail to meet criteria for ongoing inpatient care. However, among the 189 children who were still inpatients two days later, only two had failed to meet admission criteria, and since there was no child who failed to meet admission criteria but succeeded in meeting criteria for ongoing care, it was not possible to calculate an odds ratio for this relationship.

When all variables were entered for multivariate analysis for the outcome of failure to meet PAEP criteria on the day of care 2 days after admission, the variables that met the forward selection criteria numbered three: admission outside of office hours, arrival by ambulance and private health insurance. On forward selection through two steps, arrival by ambulance attained marginal significance, and private health insurance was eliminated (Table 7).

**Table 7:** Logistic Regression Model for Failure to Meet PAEP Criteria for Ongoing Care 2 Days After Admission

**Predictor Variable**	**OR**	**95% CI**	***P***
Admission out of hours (18.00 pm – 07.59 am)	0.291	0.103–0.826	.020
Arrival for admission by ambulance	3.790	0.978–14.706	.054

Abbreviations: OR, Odds ratio; PAEP, paediatric appropriateness evaluation protocol.

To determine how to avoid inappropriate length of stay in hospital, the assessors recorded what the 27 patients outside PAEP criteria were waiting for (Table 8).

**Table 8:** What Delayed Discharges Were Waiting for, as Determined by Assessors^a^

**Reason for Delayed Discharge**	**n**	**%**
A. Diagnostic/therapeutic		
Investigations or results	7	25.9
Consultant decision to discharge	6	22.2
Transfer to another acute facility	3	11.1
Review/assessment by other consultant	2	7.4
Review/assessment by other healthcare professional	1	3.7
Effective post-operative analgesia	1	3.7
B. Social/psychological		
Parental agreement	4	14.8
Community services consultation	4	14.8
Court order	1	3.7
Total	29	107.4

^a^Categories were not mutually exclusive; discharge might be delayed for a combination of reasons. Percentages are calculated using number of patients (27) as denominator.

Paediatricians’ views were attained for 21/27 (77.8%) of these patients. The consultants agreed that 7/21 (33.3%) did not warrant continuing inpatient care. Two had been admitted on a Friday evening under a care order because of fears for their safety, and another had been kept in hospital because his/her parent had been admitted acutely ill. (All three of these were taken into emergency foster care the following Monday.) Another, diagnosed with non-accidental injury (NAI), was awaiting a court order to be discharged into foster care. One child had been admitted following an episode of deliberate self-harm. Two had been admitted due to parental anxiety. For the remaining 14/21 (66.7%) patients, consultants mentioned diagnostic/therapeutic reasons to justify continuing inpatient care for 11/14 (78.6%), and/or parental anxiety, ie, social/psychological factors, for 5/14 (35.7%).

### Hospital Activity

#### Bed Capacity and Bed Occupancy

The mean bed occupancy rate during the weeks of the review was 84.1% (95% CI: 81.6%–86.5%), reaching 100% on two consecutive days in February (Table 9).

**Table 9:** Bed Occupancy During the Periods of the Study

**Date**	**Rainbow**		**PHDU**		**Sunshine**		**Total**	
	**Beds Open**	**Occupied**	**Beds Open**	**Occupied**	**Beds Open**	**Occupied**	**Beds Open**	**Occupied**	**% Occupancy**
Winter season									
Mon 18 Feb	17	11	2	2	27	20	46	33	71.7
Tue 19 Feb	17	15	2	2	27	27	46	44	95.7
Wed 20 Feb	19	19	1	1	27	20	47	40	85.1
Thu 21 Feb	17	15	2	2	27	24	46	41	89.1
Fri 22 Feb	18	18	2	2	27	26	47	46	97.9
Sat 23 Feb	23	18	0	0	27	21	50	39	78.0
Sun 24 Feb	23	12	0	0	27	18	50	30	60.0
Mon 25 Feb	23	15	0	0	27	22	50	37	74.0
Tue 26 Feb	19	19	1	1	27	27	47	47	100.0
Wed 27 Feb	19	19	1	1	27	27	47	47	100.0
Thu 28 Feb	17	17	1	1	27	20	45	38	84.4
Subtotal	212	178	12	12	297	252	521	442	84.8
Summer season									
Mon 27 May	23	22	0	0	27	23	50	45	90.0
Tue 28 May	23	20	0	0	27	26	50	46	90.0
Wed 29 May	23	20	0	0	27	26	50	46	92.0
Thu 30 May	17	18	1	1	27	25	45	44	97.8
Fri 31 May	23	21	0	0	27	24	50	45	90.0
Sat 1 June	23	13	0	0	27	23	50	36	72.0
Sun 2 June	23	12	0	0	27	12	50	24	48.0
Subtotal	155	126	1	1	189	159	345	286	82.9
Grand total	367	304	13	13	486	411	866	728	84.1

Abbreviation: PHDU, paediatric high-dependency unit.

In both winter and summer seasons, bed occupancy declined from Friday to Saturday and reached its lowest on a Sunday: 60.0% on 24 February and 48.0% on 2 June.

#### Admissions for Elective Surgery

Seventeen children were admitted for elective surgery during the study periods, all for Otorhinolaryngology. Of these, 14/17 (82.4%) followed the day of surgery admissions (DOSA) guidelines, which recommend same-day admission for elective surgery, while 3/17 (17.6%) were admitted the day before.

## Day Ward Activity

In all, 294 patients were seen on Caterpillar Day Ward during the study periods, 190/294 (64.6%) during winter season and 104/294 (35.4%) during summer season. Of these, 136/294 (46.3%) attended for phlebotomy, 45/294 (15.3%) for day-case procedures, 40/294 (13.6%) for clinical review, 30/294 (10.2%) for oncology services, 31/294 (10.5%) for GP’s rapid-access clinic (GPRAC), 12/294 (4.1%) for review of oxygen saturation and weight check, and 5/294 (1.7%) for other reasons. Clinical reviews included infusions, general and subspecialty assessments, and post-discharge follow-up, as well as referrals to the GPRAC. Of 39 children referred to GPRAC, 31/39 (79.5%) were assessed on the day ward and 11/39 (28.2%) were admitted.

## Discussion

Childhood morbidity consumes a substantial portion of healthcare resources in terms of hospital bed utilisation.^15^ In addition, high levels of bed occupancy give rise to problems of impaired access, poorer patient outcomes, and increased stress among provider staff.^1^ In a cross-sectional analysis of the impacts of bed occupancy on ED care in a tertiary care children’s hospital in the United States, Hillier et al found that, when bed occupancy levels exceed 80%, every additional 5% increase in occupancy was associated with an increased delay of 34 minutes for admitted patients, and the increasing occupancy led to increasing odds of leaving without being seen or of being treated in a hallway bed.^16^ Assuming that this kind of influence on patient flow applies internationally, all practicable measures must be taken to identify and address avoidable influences on high bed occupancy.

A number of international bed utilisation studies used the original or modified versions of the PAEP to investigate inappropriate bed utilisation, either at the time of admission or on subsequent days of care. These found that the rate of inappropriate bed utilisation ranged from 2.0% to 40.7% at the time of admission to hospitals in Israel,^9^ Italy,^10,11^ Kuwait,^12^ Australia,^6^ the United Kingdom and the United States,^7^ and from 21.4% to 55.5% on subsequent days of care in the United States,^13^ Canada,^14^ and Italy.^10,11^ Thus, the observed percentages of 3.6% PAEP-inappropriate admissions and 14.3% PAEP-inappropriate episodes of ongoing care in this Irish RPU lie towards the lower end of the scale by international standards.

The percentages that failed to meet PAEP criteria in this study compare favourably with those in the other most comparable Irish study: An unpublished study in two general hospitals in the Dublin North-East area in 2008 (Downey and Bedford, 2008) found that 9.8%–10.2% of paediatric patients failed to meet criteria on the DOA, and 19.6%–34.7% failed to meet criteria on the day of care. A British study using the same version of the PAEP found that 8.4% of paediatric admissions to 13 hospitals in England in 1990–1991 failed to meet admission criteria.^8^ The observed figures do not match those of another unpublished study conducted in three secondary/tertiary care children’s hospitals in Dublin in 2010 (Feely, 2010), which found that none of the paediatric patients failed to meet admission criteria, and that 0.0%–6.4% failed to meet day-of-care criteria. However, 30% of patients in Dublin paediatric hospitals are referred from other hospitals, while the RPU in Limerick serves the adjacent urban area and surrounds, including significant areas of deprivation. Similar differences in proportions of patients failing to meet PAEP criteria were found in a study of three paediatric hospitals with different referral functions in Louisiana.^7^

The fact that hypothesis testing in this study failed to identify attributes of the children or of the circumstances of their care as potential predictors of inappropriate admission likewise suggests high standards in admission processes. Multivariate analysis of Vincitorio and colleagues’ sample of 429 inpatients at an Italian children’s hospital,^11^ and of Bianco and colleagues’ sample of 656 inpatients at another,^10^ indicated preferential admission of children who lived at a greater distance from the hospital, or of those who arrived by day, respectively, and bivariate analysis of Katz and colleagues’ sample of 221 children at a hospital in Israel indicated preferential admission of children aged >1 year, of Jewish ethnicity and/or self-referred^9^; no such preference was observed on bivariate or multivariate analysis of this Irish sample of 355 admissions. Similarly, multivariate analysis of Bianco et al indicated a tendency towards inappropriate admission by medical vs. surgical specialty^10^; again, no such preference was observed on multivariate analysis of this Irish sample. While the somewhat smaller sample size of this Irish sample relative to the size of the two Italian studies may have resulted in less statistical power on conducting multivariate analysis, the achievement of very low proportions of inappropriate admissions gives rise to a requirement for even greater statistical power to detect any predictive associations that might exist.

The identified associations between circumstances of admission and success or failure to meet PAEP criteria for ongoing care two days later merit further investigation. Admission out-of-hours may serve as an indicator of severity of illness, extending 2 days beyond the point of admission. Conversely, arrival for admission by ambulance may serve as an indicator of a lack of parental self-efficacy and/or increased expectations of services, and this may suggest a need to address parental anxiety and to promote self-efficacy.

Admission or assessment on a Saturday or Sunday was not associated with any difference in success or failure to meet the PAEP criteria, despite the fact that bed occupancy declined at the weekend, reaching its trough on Sunday. This is consistent with aforementioned research from Italy, which likewise observed no association between weekend or weekday admission and success or failure to meet PAEP criteria.^10,11^ The provision of consultant cover to conduct regular ward rounds at the RPU over weekends may minimise differences between weekday and weekend patterns of admission and discharge.

On the whole, the achievement of 96.6% of admissions meeting PAEP admission criteria and of 85.7% of inpatients meeting criteria for ongoing care 2 days after admission is to be commended. Factors contributing to this success are thought to include the employment of appropriately skilled NCHDs, the review of all admissions on a daily basis (including weekends) during ward rounds involving admitting doctors, nurses and consultants and the provision of strong continuing medical education.

Despite the modest proportions involved, the reasons for inappropriate admissions and for episodes of ongoing inpatient care merit attention.

Because parental anxiety is such a prominent factor in inappropriate inpatient care, approaches to address it merit consideration. If health literacy interventions for parents are effective in reducing children’s ED utilisation, as indicated by systematic review of Morrison et al,^17^ they may also reduce inpatient care.

The number of children admitted for lack of another place of safety (five) and the number of these in continuing inpatient care after 2 days (three) give rise to concern. Although an acute hospital may be safer than a home environment where a child risks NAI, it bears a risk of nosocomial infection. Even more salient is the question of appropriate facilities for one excess admission and one delayed discharge that arose solely because a parent had been admitted, as these two children were neither ill nor in danger of abuse.

Those patients who failed to meet the criteria for diagnostic/therapeutic reasons provide indications for the development of relevant services, eg, for the management of constipation in the community and for out-of-hours investigations. A needs assessment and an economic evaluation may be required to explore this aspect.

The Irish health services permit private patients to be treated in public hospitals. A private patient pays the treating consultant (through the insurance company) for services provided, along with additional charges of up to €1000 per night to the hospital treatment (through the insurance company) in a bed in the public hospital.^18,19^ These arrangements have raised concerns that hospital care might be influenced by private health insurance.^20^ However, with respect to the appropriateness of both admission and ongoing care, our study found no statistically significant difference between public and private paediatric patients.

Consistent with bed occupancy rates in the RPU that often exceeded 90% (Table 9), the Organisation for Economic Co-operation and Development’s (OECD’s) most recent report regarding healthcare activities indicate that Ireland had the third highest rate, at over 90% – significantly higher than the 85% considered the safe limit in countries including the United Kingdom.^21^ A Health Service Executive (HSE)-commissioned review of acute bed capacity requirements until the year 2020 notes that high utilisation may cause delays in admissions requiring acute services, and that overcrowding may increase the risk of hospital-acquired infections.^22^ Thus, the high bed occupancy rates underline the need to provide enhanced services in the community.

The 9.9% of admissions whose only PAEP criterion for admission was a requirement for intravenous fluids or injections merit closer scrutiny. Doré-Bergeron et al found short-term, intravenous antibiotic therapy at a day treatment centre feasible for the management of infants with presumed febrile urinary tract infections.^23^

The international literature indicates potential advantages in developing the GPRAC as an acute assessment service to filter admissions. Studies at English district general hospitals have found that assessment services extending into late evening may reduce the numbers of children admitted as emergency overnight admissions.^24,25^ However, other authors caution that this approach may incur similar expense as overnight admission,^26^ or incur an increased financial burden on primary healthcare teams,^27^ so such developments should be subject to cost-effectiveness analysis.

Arising out of the findings of this study, the RPU is evaluating extension of GPRAC services, optimising DOSA practice recommendations, proposing a paediatric acute medical assessment unit (PAMU) and devising new initiatives such as outpatient paediatric antimicrobial therapy (OPAT) for appropriate patients. Recommendation to increase the approved bed capacity of paediatric high-dependency unit (PHDU) from two to four has been submitted. On the initiative of the Maternal and Child Health Directorate and hospital’s executive, a dedicated new six-bed paediatric section of the ED, with audio-visual separation from the adult section, has been developed. This facility has the potential to develop as a full-scale PAMU subject to the availability of additional resources. Re-audit and evaluation are planned on completion of service developments, to measure performance and to generate sustained improvements.

## Limitations

While the PAEP criteria used as the audit standard for this study have been developed for use in the United Kingdom by modification of the US criteria and have undergone formal testing to show high inter-rater reliability, they have not undergone similar testing in Ireland. However, because paediatric and public-health specialists have previously deemed these criteria acceptable for use in Irish hospitals, as documented in the unpublished reports mentioned above (Downey and Bedford, 2008; Feely, 2010), it has been considered suitable for the conduct of this study also. The consultant paediatricians at the RPU reviewed and approved the criteria in advance, and agreed with the assessors that the instances of “inappropriate” admissions or ongoing care merited closer scrutiny.

Although the dual sampling strategy of this study (winter and summer) increases its representativeness and validity, it may have failed to capture changes in behaviour during periods of unusual activity, eg, the Christmas season, or at times of changes in NCHDs in Ireland, in early January and early July. Because of the relatively modest percentages of cases failing to meet PAEP criteria, the study may not have been powerful enough to detect statistically significant associations between potential predictors and the outcomes of interest, particularly failure to meet admission criteria.

While this study has identified a number of admissions and instances of ongoing inpatient care that, according to PAEP criteria, should more appropriately be managed elsewhere, it does not include a systematic survey of gaps in services or any economic analysis for the provision of additional services that may be required. In some respects, the feasibility of providing services for paediatric inpatients must be considered in the context of the broader hospital setting; for example, the provision of diagnostic facilities at weekends must consider the needs of adult patients also. This is considered to be beyond the scope of the present study.

## Conclusion

Although this RPU performed very well with respect to the percentages of admissions that met PAEP criteria, the results suggest some room for improvements regarding:

- Emergency access to social services and foster care
- Management of parental anxiety
- Provision of key investigations and timely availability of results at weekends and in outpatient settings
- Child health support services in the community.

Admission of some children the day before elective surgical procedures presents an unacceptable risk for nosocomial infection as well as avoidable utilisation of acute service resources.

Potential ways to provide ambulatory treatment to some of the almost one-tenth of all admissions where the only PAEP criterion met was the requirement for intravenous therapy, in an appropriately resourced facility attached to ED or in the day ward, should be explored.

This study of bed utilisation and activity at a RPU in a University hospital setting could be reproduced to understand and improve the structure and processes of paediatric acute care and patient flow in similar settings in other European countries.

## Acknowledgements

The authors would like to thank the nursing, administrative and clinical staff of the RPU, the administrative staff of Healthcare Records, UHL, Limerick, Ireland and the administrative staff at the Department of Public Health, Limerick, Ireland. Special thanks are due to Ms. Maura Fitzgerald, Deputy Director of Nursing, UHL, Limerick, Ireland Dr. John Twomey, Consultant Paediatrician, UHL, Limerick, Ireland, Dr. Siobhán Gallagher, Consultant Community Paediatrician, UHL, Limerick, Ireland and Ms. Hilary Cowley, Research Officer, Department of Public Health, Limerick, Ireland.

## Ethical issues

The protocol was approved by UHL Audit Committee, UL Hospitals, Limerick, Ireland.

## Competing interests

The authors declare that they have no competing interests.

## Authors’ contributions

CÓ participated in designing the study, gathering data, analysing, and interpreting data and writing the manuscript. MM participated in designing the study, gathering data, interpreting data, and critical revision of the manuscript. JS participated in designing the study, analysing data, and critical revision of the manuscript. RP participated in designing and supervising of the study and critical revision of the manuscript. RP also acted as the owner of the audit and project lead.

## Authors’ affiliations

^1^Department of Public Health, Health Service Executive, Dublin, Ireland. ^2^Statistical Consulting Unit, University of Limerick, Limerick, Ireland. ^3^Regional Paediatric Unit (Children’s Ark), University Hospital Limerick (UHL), Limerick, Ireland.

## Key messages

**Implications for policy makers**

- Efficient triage and ambulatory treatment practices would reduce the rate of inappropriate admissions to a Regional Paediatric Unit (RPU) to 3.4%, even with a mean bed occupancy rate of 84.1%.
- Close scrutiny of reasons for admission may identify issues such as parental anxiety that contribute to inappropriate inpatient care, even without statistical significance.
- Emergency access to social services and foster care could prevent hospital admission of otherwise healthy children.
- The provision of investigations and timely availability of results at weekends and in outpatient settings may shorten length of stay for some children.
- As the administration of intravenous medications or fluids alone contributes to approximately one in ten paediatric admissions, options to provide these without overnight admission may save acute hospital beds for other, sicker patients.

**Implications for public**

We compared the reasons for admission and ongoing inpatient care of children in a regional hospital with a list of recognised reasons why overnight care should be necessary. Very few admissions were unnecessary, however, some children were kept in hospital longer than recommended because their parents were afraid they could not adequately care for them at home, and some were kept in because foster care could not be arranged over the weekend. The results suggest that doctors and managers can do more to develop community-based services to help and support parents to care for their sick children at home whenever possible. Improved services to provide fluids and antibiotics in suitable facilities without overnight admission may allow children to go home sooner, thus, relieving over-crowding and reducing the risk of children catching infectious diseases in hospital. may allow children to go home sooner, thus, relieving over-crowding and reducing the risk of children catching infectious diseases in hospital.
